# Lense–Thirring precession after a supermassive black hole disrupts a star

**DOI:** 10.1038/s41586-024-07433-w

**Published:** 2024-05-22

**Authors:** Dheeraj R. Pasham, Michal Zajaček, C. J. Nixon, Eric R. Coughlin, Marzena Śniegowska, Agnieszka Janiuk, Bożena Czerny, Thomas Wevers, Muryel Guolo, Yukta Ajay, Michael Loewenstein

**Affiliations:** 1 Kavli Institute for Astrophysics and Space Research, Massachusetts Institute of Technology, Cambridge, MA USA; 2 Department of Theoretical Physics and Astrophysics, Masaryk University, Brno, Czech Republic; 3 School of Physics and Astronomy, University of Leeds, Leeds, UK; 4 Department of Physics, Syracuse University, Syracuse, NY USA; 5 School of Physics and Astronomy, Tel Aviv University, Tel Aviv, Israel; 6 Center for Theoretical Physics, Polish Academy of Sciences, Warsaw, Poland; 7 Space Telescope Science Institute, Baltimore, MD USA; 8 European Southern Observatory, Santiago, Chile; 9 Department of Physics and Astronomy, Johns Hopkins University, Baltimore, MD USA; 10 Department of Astronomy, University of Maryland, College Park, MD USA; 11 Center for Research and Exploration in Space Science and Technology, NASA Goddard Space Flight Center, Greenbelt, MD USA; 12 NASA Goddard Space Flight Center, Greenbelt, MD USA

**Keywords:** General relativity and gravity, Compact astrophysical objects, Transient astrophysical phenomena, High-energy astrophysics

## Abstract

An accretion disk formed around a supermassive black hole after it disrupts a star is expected to be initially misaligned with respect to the equatorial plane of the black hole. This misalignment induces relativistic torques (the Lense–Thirring effect) on the disk, causing the disk to precess at early times, whereas at late times the disk aligns with the black hole and precession terminates^1,2^. Here we report, using high-cadence X-ray monitoring observations of a tidal disruption event (TDE), the discovery of strong, quasi-periodic X-ray flux and temperature modulations. These X-ray modulations are separated by roughly 15 days and persist for about 130 days during the early phase of the TDE. Lense–Thirring precession of the accretion flow can produce this X-ray variability, but other physical mechanisms, such as the radiation-pressure instability^3,4^, cannot be ruled out. Assuming typical TDE parameters, that is, a solar-like star with the resulting disk extending at most to the so-called circularization radius, and that the disk precesses as a rigid body, we constrain the disrupting dimensionless spin parameter of the black hole to be 0.05 ≲ ∣*a*∣ ≲ 0.5.

## Abstract (web summary)

The accretion disk from a star tidally disrupted by a supermassive black hole undergoes Lense–Thirring precession with strong, quasi-periodic X-ray flux and temperature modulations.

## Main

**Extended Data Fig. 1:**
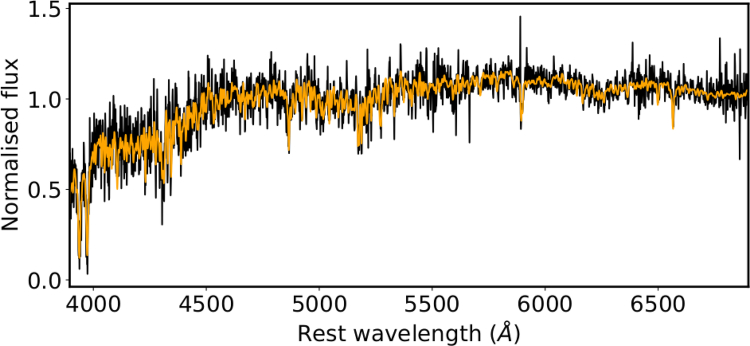
SDSS host spectrum of AT2020ocn (black). Overlaid in orange is the best fit MILES model template obtained through pPXF fitting, broadened to a velocity dispersion of 82 ± 4 km s^−1^. No narrow emission lines are evident in the spectrum.

AT2020ocn/ZTF18aakelin is an optical transient from the centre of a previously quiescent galaxy at a redshift of 0.0705 (ref. ^5^) (Extended Data Fig. 1). Follow-up optical spectra taken 1–2 months post-discovery showed a blue continuum and a broad He II line (see figure 16 of ref. ^6^). On the basis of these properties, it was classified as a tidal disruption event (TDE)^5,6^. We measured the stellar velocity dispersion of the host galaxy using an optical spectrum taken 12 years before the outburst (see section ‘Black hole mass from the stellar velocity dispersion of the host galaxy’). Assuming the scaling relation between the host stellar velocity dispersion and black hole mass implies a disrupting supermassive black hole (SMBH) mass of 10^6.4±0.6^*M*_⊙_, in which the error bar includes both the measurement and the systematic uncertainties in the scaling relation.

**Fig. 1:**
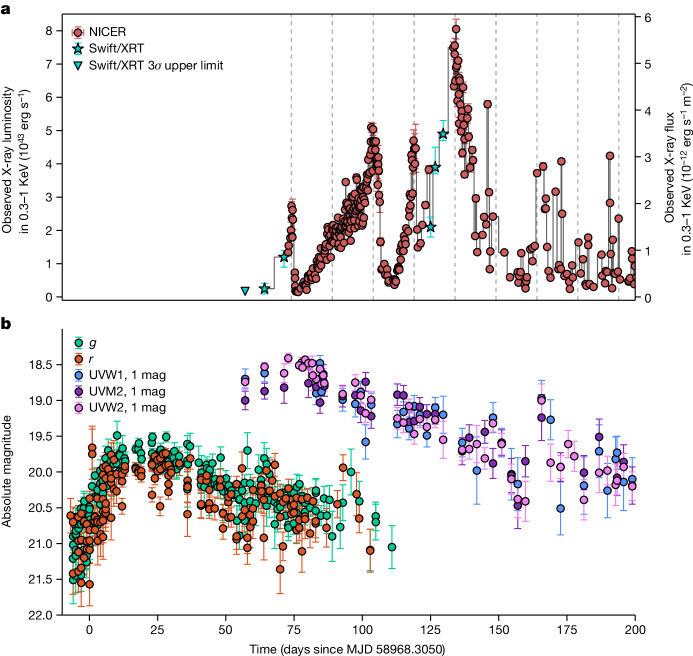
Multi-wavelength evolution of AT2020ocn. **a**, X-ray luminosity (0.3–1.0 keV) versus time since optical discovery. Gaps in NICER monitoring are filled by Swift data. The dashed and vertical lines are separated by 15 days to guide the eye. Archival Swift X-ray (0.3–1.0 keV) 3*σ* upper limits from before MJD 58274 is 3 × 10^−14^ erg s^−1^ cm^−2^ (4 × 10^41^ erg s^−1^). The first X-ray or XRT data point is a non-detection with a 3*σ* upper limit of 1.7 × 10^−13^ erg s^−1^ cm^−2^. **b**, Optical and UV evolution of AT2020ocn. All values are host-subtracted. All the other error bars represent 1*σ* uncertainties. See ‘Data availability’ section below to access the data.

**Extended Data Fig. 2:**
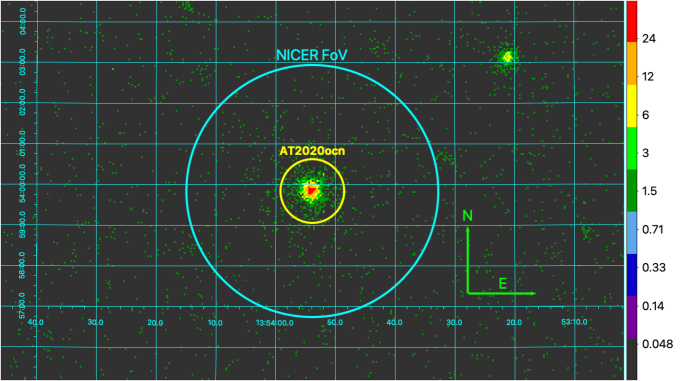
*Swift*/XRT 0.3-1.0 keV image of *NICER*’s FoV. The yellow circle with a radius of 47″ and is centered on AT2020ocn’s optical coordinates of 13:53:53.803, +53:59:49.57 (J2000.0 epoch). The outer cyan circle shows *NICER*/XTI’s approximate field of view of 3.1′ radius. There are no contaminating sources within *NICER*’s field of view. The north and east arrows are each 100″ long. The colorbar shows the number of X-ray counts.

Roughly a day after the TDE classification, the Neutron Star Interior Composition Explorer (NICER) started a high-cadence (multiple visits per day) monitoring program on Modified Julian Date (MJD) 59041. Here we focus on approximately the first 4 months of monitoring data during which multiple soft X-ray (0.3–1.0 keV) flares are evident (Fig. 1a and Extended Data Fig. 2). A visual inspection suggests that they are regularly spaced, roughly 15 days apart, and similar modulations are not present in the optical–ultraviolet (UV) bands (Fig. 1b).

**Fig. 2:**
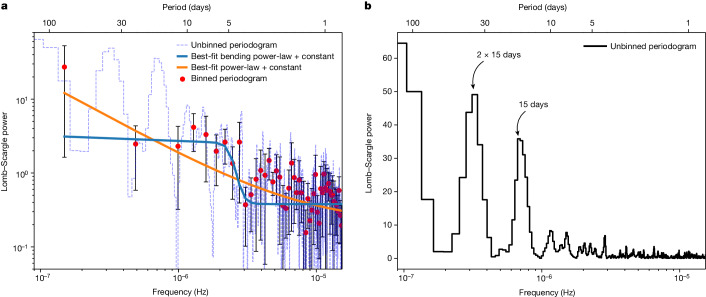
LSP of the observed 0.3–1.0 keV NICER light curve. **a**, The blue dashed histogram represents the unbinned LSP, whereas the red data points are the binned LSP excluding the peaks near 15 days and 2 × 15 days. The solid blue and orange curves are the best-fit bending power-law + constant and power-law + constant models, respectively, used to characterize the noise continuum. **b**, Unbinned periodogram (same as **a** but the *y*-axis is shown on linear scale without overplotting the continuum for clarity).

**Extended Data Fig. 3:**
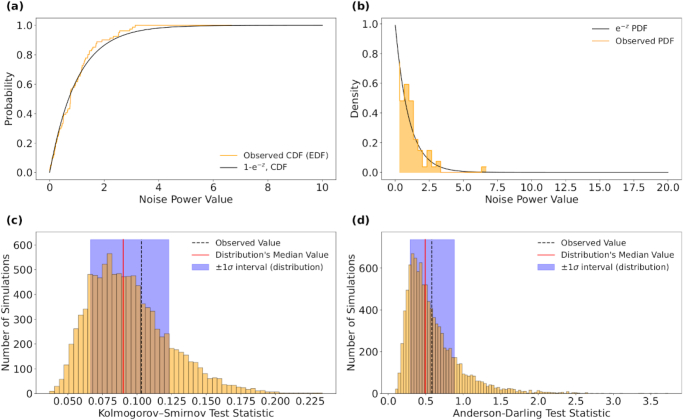
White noise tests for the distribution of noise powers in the observed Lomb Scargle periodogram (LSP) using power values that correspond to timescales slower than 3 days and excluding bins near 15, 2 × 15 and 4 × 15 days. **(a) Comparison of the cumulative distribution functions (CDFs) of the observed noise powers and the expected exponential distribution**. The CDF of the observed LSP of the observed light curve is represented by the orange histogram, while the solid black line shows the CDF of white noise powers, which follows an exponential distribution. **(b) Comparison of the probability density functions of the observed noise powers and the expected exponential distribution**. The solid black line is the PDF of white noise distribution, whereas the shaded orange histogram represents the PDF of the observed noise powers in the LSP of the observed light curve. **(c) Distribution of the Kolmogorov-Smirnov test statistic derived from simulations**. **(d) Distribution of the Anderson-Darling test statistic using simulations**. In both **(c)** and **(d)**, the solid red and the dashed black lines represent the median of the distribution and the observed value, respectively and the shaded blue regions indicate the ± 1*σ* values of their respective distributions. The null hypothesis that the noise powers are exponentially distributed, i.e., consistent with white noise, cannot be rejected.

**Extended Data Fig. 4:**
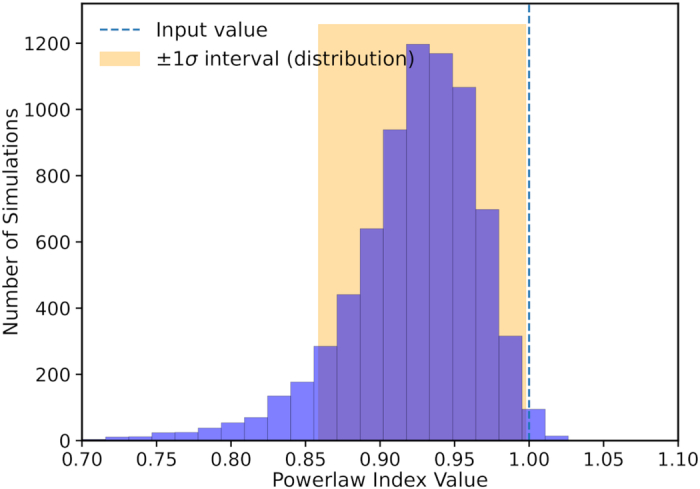
Check for powerlaw index bias due to uneven sampling. The distribution represents the powerlaw index values of the simulated LSPs with an input index of 1.0.

**Extended Data Fig. 5:**
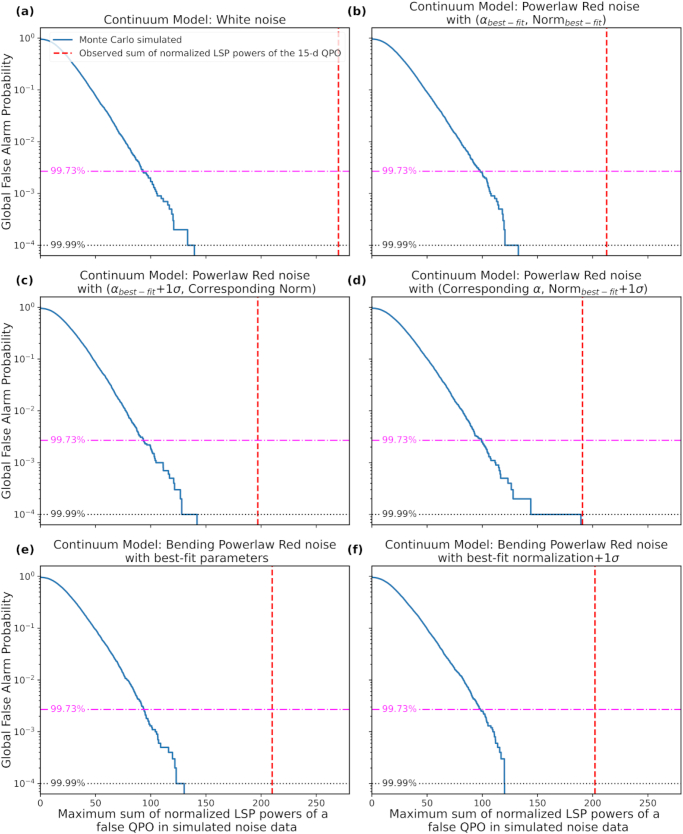
Estimates of the global false alarm probability (FAP) of finding a broad peak in the simulated LSPs. Each panel is for a different underlying noise continuum: (a) white noise, (b) powerlaw red noise with best-fit powerlaw index, *α*_*best*−*fit*_, and normalization, Norm_*best*−*fit*_, (c) powerlaw red noise with best-fit powerlaw index + 1*σ* and its corresponding normalization, (d) powerlaw red noise best-fit normalization + 1*σ* and its corresponding powerlaw index, (e) bending powerlaw red noise with best-fit parameters, and (f) bending powerlaw red noise with best-fit normalization + 1*σ* and corresponding parameters. The dashed, vertical line in each panel represent the observed QPO at 15-days.

To quantify the variability, we extracted a Lomb–Scargle periodogram (LSP) (refs. ^7,8^) of the background-subtracted 0.3–1.0 keV count rate (Fig. 2). As expected, there is a broad peak at ${17}_{-2.4}^{+1.2}\,{\rm{days}}$ with additional integral harmonics. The uncertainty represents the full width at half maximum of the highest bin near 17 days. Given this measurement uncertainty and the fact that the individual peaks in Fig. 1a seem better aligned with the 15-day vertical lines, we refer to this signal as the 15-day quasi-periodicity. Using a rigorous set of Monte Carlo simulations, we estimate the global statistical significance (false alarm probability (FAP)) of finding a broad peak as strong as the one found in the data by chance to be less than 1 in 10,000 (or >3.9*σ* for all the continuum models considered, assuming a Gaussian distribution; see section ‘Estimating the statistical significance of the 15-day X-ray flux modulations’ and Extended Data Figs. 3–5). Our global FAP estimate (1) was tested against a range of underlying noise continuum models; (2) included a search over all frequencies and periods sampled by the LSP (1–100 days); and (3) included a search for broad peaks in the noise LSPs with a wide range of coherence values from 2 to 10. Coherence is defined as the ratio of the centroid frequency of a broad power spectral peak over its width, and X-ray quasi-periodic oscillations (QPOs) seen in accreting stellar-mass black holes typically have values below 10 (ref. ^9^).

**Fig. 3:**
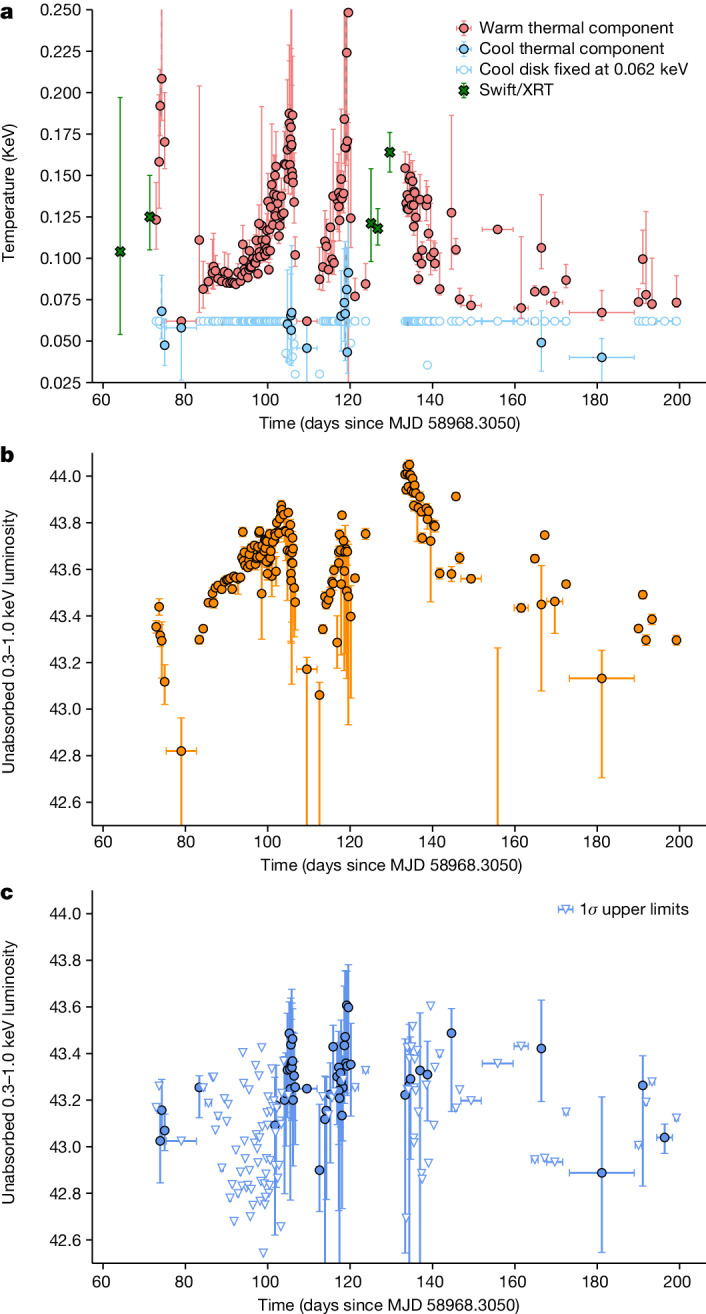
X-ray spectral evolution of AT2020ocn. **a**, Temperature evolution of the warm and the cool X-ray thermal components. When the cool component was not statistically required by the data, we froze its temperature to 0.062 keV and computed the 1*σ* upper limit on the flux of the cool component. **b**,**c**, Logarithm of luminosity of the warm component (**b**) and the cool component (**c**). All error bars represent 90% uncertainties.

We then performed time-resolved X-ray spectral analysis on the first 130 days of NICER data. Our main findings are that (1) the X-ray spectrum of AT2020ocn is soft and can be described by two thermal components: a cool component and a warm component; (2) the overall X-ray flux shows quasi-periodic modulations that repeat roughly every 15 days; and (3) these modulations are accompanied by X-ray temperature modulations on the same timescale (Fig. 3b).

We consider a range of models, including a repeating partial TDE^10^, repeated debris stream self-interactions^11,12^ and neutral column density changes and disfavour them based on the observed properties of AT2020ocn (Methods). Radiation-pressure instability (RPI) (refs. ^3,4^) can reproduce the approximately 15-day timescale if the outer disk is truncated at roughly 30 gravitational radii, *R*_g_. However, it predicts a modulation amplitude that is more than two orders of magnitude larger than that observed in Fig. 1a. These amplitudes can be damped to levels comparable to observations if a magnetic field of about 10^4^ G is present in the inner disk. Thus, RPI can, in principle, explain the X-ray modulations but requires fine-tuning of multiple parameters (see section ‘Discussion of various models’).

In a TDE, the stellar orbit and the SMBH spin axis will be misaligned, and the disk material undergoes Lense–Thirring precession in which the plane of the orbit precesses around the spin vector of the black hole. Emission from the central accretion disk, combined with Lense–Thirring precession of the disk, may provide a straightforward explanation for the soft X-ray spectrum, flux and temperature modulations, and the lack of similar modulations in the optical–UV bands. Lense–Thirring precession is commonly accepted to be the cause of some X-ray QPOs of the order of seconds from accreting stellar-mass compact objects^13–15^. The shape of the outbursts of AT2020ocn are similar to some of those exhibited by the X-ray binaries GRS 1915+105 and IGR J17091-3624. These systems show quasi-periodically repeating state cycles^16–18^, and this behaviour has often been interpreted as evidence for RPI^4,19^, but it has also been suggested that it may arise from Lense–Thirring precession of a radially narrow region of the disk close to the black hole horizon^20^. We, therefore, consider Lense–Thirring precession of a newly formed accretion disk around an SMBH. This can manifest in two modes: (1) if the accretion rate is sufficiently high, and thus the disk is geometrically thick such that the disk angular semi-thickness *H*/*R* is larger than the disk viscosity parameter *α* ≈ 0.1 (ref. ^21^), then the precession can be efficiently communicated by pressure waves^22^. This allows a substantial portion of the disk to precess as a rigid body^1,2^; or (2) for lower accretion rates, in which the disk is thin, the inner disk can tear into discrete annuli that precess individually^23^. In the former case, the observed X-ray modulations are a result of the changing orientation of the system, whereas in the latter scenario, the X-ray modulations result from a combination of changing orientation and accretion of discrete precessing annuli. We focus on the first mode here because the accretion rate is, in general, expected to be high following a TDE (for example, figure 1 of ref. ^24^), and discuss the second mode in the Methods.

When the disk precesses as a rigid body, both the observed flux and the disk temperature can exhibit modulation over the precession period (for example, see figure 4 of ref. ^25^). This is because, during certain precession phases, our line of sight enables us to observe the hot inner disk, whereas during the other phases, our view of the inner disk becomes obstructed, allowing mostly the cool outer disk to be visible (Extended Data Fig. 7). Also, Fig. 3c suggests that the variability of the cool component roughly traces the warm component—albeit with large error bars. This is consistent with the precession of an extended disk rather than a narrow ring. Although the origin of optical–UV emission from TDEs is still debated, it is generally not thought to be direct disk emission. Some models include stream–stream collisions^11^ and X-ray reprocessing^26,27^. Thus, a precessing inner disk that produces soft X-rays should not modulate the optical–UV flux originating far away from the hole. Furthermore, theoretical studies have estimated that, for SMBH weighing 10^5^–10^7^*M*_⊙_, this rigid body precession should last between 0.4 years and 0.7 years before the accretion rate declines to the point that rigid precession is no longer possible and the disk aligns with the black hole spin (see figure 2 of ref. ^1^). This is consistent with the X-ray modulations lifetime of AT2020ocn of about 130 days (Fig. 1a). The first few X-ray flares of AT2020ocn are asymmetric, and this can be interpreted as a precessing disk that is warped, rather than planar, which can lead to an abrupt obscuration of the inner hot gas. Also, the second peak in the total X-ray flux (near 90 days in Fig. 1a) appears blended with the third peak. However, it is more pronounced in the luminosity of the warm component and temperature evolution (Fig. 3a,b). We can explain this in the precession model if the disk initially has a significant geometric thickness. This can shield the inner warm material more when compared with other time periods.

**Fig. 4:**
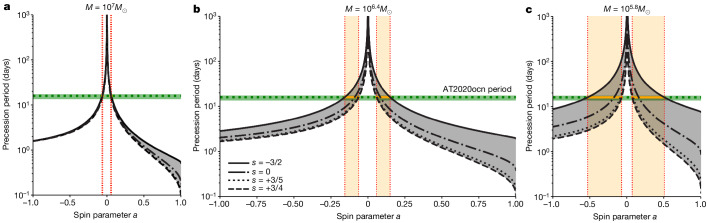
Spin constraints on SMBH of AT2020ocn based on the rigid body Lense–Thirring precession model. **a**–**c**, The precession period–spin relations for the upper limit (**a**), best fit (**b**) and the lower limit (**c**) on the SMBH mass inferred from the *M*–σ_*_ relation (see section ‘Black hole mass from the stellar velocity dispersion of the host galaxy’). For these calculations, we assumed standard TDE parameters, that is, a solar-like star with the resulting disk extending out to tidal radius. The grey-shaded area captures the uncertainty arising from various disk–density profiles. The green dashed horizontal line represents the rest-frame period of AT2020ocn (15.9 days) and the green band reflects the uncertainty in the rest-frame period (${15.9}_{-2.2}^{+1.1}\,{\rm{days}}$). For a given SMBH mass, the possible spin values are shown by the orange-shaded area. The overall spin constraint for the SMBH of AT2020ocn across all masses is 0.05 ≲ ∣*a*∣ ≲ 0.5 (see Extended Data Table 1 for specific values).

Assuming rigid body precession is the origin of this 15-day modulation and standard TDE parameters, we constrain the spin parameter to be 0.05 ≲ ∣*a*∣ ≲ 0.5 (see Fig. 4 and section ‘Spin estimate assuming rigid body precession’ for details).

The accretion of stellar debris following a TDE can be highly chaotic owing to various physical effects, and this is evident in the plethora of low-cadence X-ray TDE light curves^28,29^. Our work demonstrates that high-cadence X-ray monitoring can show regular processes happening amongst the chaos from the highly relativistic regions near disrupting SMBHs. If regular modulations such as the ones identified here are caused by rigid body disk precession, this enables an independent avenue to measure the SMBH spins. It is interesting to note that the upcoming all-sky surveys such as the Rubin observatory could detect hundreds of TDEs per year (ref. ^30^). Even if only a fraction of them show early X-rays and quasi-periodic precession-like modulations, this could result in independent constraints on SMBH spin distribution in the local Universe.

## Methods

### Observations and data analysis

We used data from the NICER (X-ray), XMM-Newton (X-ray), Swift (X-ray and UV), the Zwicky Transient Facility (ZTF; optical)^31^ and Sloan Digital Sky Survey (SDSS; optical). In the following sections, we describe these data and their reduction procedures. Throughout, we adopt a standard ΛCDM cosmology with *H*_0_ = 67.4 km s^−1^ Mpc^−1^, *Ω*_*m*_ = 0.315 and Ω_*Λ*_ = 1 − Ω_*m*_ = 0.685 (ref. ^32^). Using the cosmology calculator of ref. ^33^, the redshift of 0.0705 of AT2020ocn corresponds to a luminosity distance of 330 Mpc.

#### X-ray data

##### NICER

NICER started monitoring AT2020ocn on 11 July 2020, roughly 10 weeks after ZTF discovered it on 29 April 2020 (MJD 58968.305) (ref. ^5^). NICER continues to monitor AT2020ocn on a daily cadence at the time of writing of this paper. However, for this work, we consider only data taken over around the first 130 days, that is, until 17 November 2020, when X-ray flares are prominent.

We started our analysis by downloading the raw, level 1, publicly available data from the HEASARC archive (https://heasarc.gsfc.nasa.gov/cgi-bin/W3Browse/w3browse.pl) and reduced them using the nicerl2 tool with the default screening filters recommended by the NICER data analysis guide (https://heasarc.gsfc.nasa.gov/docs/nicer/analysis_threads/nicerl2/). The resulting good time intervals (GTIs), that is, uninterrupted data segments, ranged from 250 s to about 2,600 s.

NICER consists of 52 co-aligned detectors (focal plane modules (FPMs)). In any given GTI, some detectors may be hot, that is, the optical light loading can make them behave anomalously. We identify these hot detectors on a per GTI basis by flagging FPMs whose 0–0.2 keV count rate is more than 3*σ* above the sigma-clipped median value of all FPMs during that GTI (see refs. ^34,35^ for more details).

Using the 3c50 background model^36^, we first extracted the source and the background spectra on a per GTI basis by excluding the appropriate hot FPMs. We then computed the background-subtracted count rates (counts s^−1^ FPM^−1^). Because the source is faint as per ref. ^36^, we also applied the so-called level-3 filtering as described in ref. ^36^. The source was typically above the estimated background in the 0.3–1.0 keV band. Therefore, we adopted this energy for most of our analysis in this paper. However, because both the source and the background are variable, in some GTIs, the background exceeded the source counts down to 0.6 keV. For these spectra, modelling was restricted to an appropriate value lower than 1 keV.

All spectra were binned using an optimal binning scheme of ref. ^37^ to have at least 25 counts per spectral bin. This was done using the ftgrouppha task of HEASoft (https://heasarc.gsfc.nasa.gov/lheasoft/help/ftgrouppha.html). We performed spectral modelling in XSPEC^38^ and Python version of XSPEC, PyXspec, using the *χ*^2^ statistic.

##### XMM-Newton

XMM-Newton observed AT2020ocn on four occasions, two taken roughly a week after NICER started monitoring the source (18 and 21 July 2020) and the other two taken 1–2 years later. Here we use only the data from EPIC-pn detector from the first two datasets with observation IDs 0863650101 (XMM#1) and 0863650201 (XMM#2). We reduced the publicly available raw data using the standard epproc tool of XMM software xmmsas v.19.1.0 with the latest calibration files. From these cleaned eventfiles, we visually inspected the 10–12 keV count rates from the entire field of view to identify epochs of background flaring. GTIs were chosen to exclude these flaring windows. Using only the events that occurred during the GTIs, we extracted source spectra (corrected for pileup) by using annuli with inner radii of 5″ and 10″ for XMM#1 and XMM#2, respectively. The annuli were centred on optical coordinates: (13:53:53.803, +53:59:49.57) J2000.0 and had an outer radius of 20″. Background spectra were extracted using events from two nearby circular regions of 50″ each. Finally, spectra were grouped using the xmmsas tool specgroup to have a minimum of 1 count per spectral bin.

##### Swift

Swift started monitoring AT2020ocn on 25 June 2020 at a much lower cadence than NICER, roughly one visit every few days. There were also 14 archival observations with a total exposure of 12.3 ks before the outburst, that is, between MJD 57255.452 (14 August 2015) and MJD 58268.978 (30 May 2018). Using the standard xrtpipeline tool, we reduced all the X-ray telescope (XRT) observations taken before 17 November 2020. Source events were extracted from a circular aperture of 47.1″ and background events were chosen from an annulus with an inner and outer radii of 70″ and 210″, respectively. We used only events with grades 0–12 as recommended by the data analysis guide. Swift data were used for three reasons: (1) to estimate an upper limit on the X-ray flux before the outburst; (2) to fill-in the NICER data gaps; and (3) to confirm that there are no contaminating sources within the field of view of NICER of AT2020ocn (Extended Data Fig. 2).

#### Optical–UV observations

##### Zwicky Transient Facility

AT2020ocn was discovered and reported by ZTF and released as a transient candidate ZTF18aakelin in the Transient Name Server^39^. We performed point spread function photometry on all publicly available ZTF data using the ZTF forced-photometry service^40^ in the *g*- and *r*-bands. We report our photometry, corrected for Galactic extinction of *A*_V_ = 0.0153 mag (ref. ^41^).

##### Swift/UVOT

We perform photometry on Swift/UVOT^42^ observations of AT2020ocn with the uvotsource task in HEASoft package v.6.29 using a 5″ aperture on the source position. Another nearby region of 40″ free of any point sources was used to estimate the background emission. The host contribution was subtracted using a modelled spectral energy distribution. Similar to ZTF data, UVOT photometry was corrected for Galactic extinction.

### Black hole mass from the stellar velocity dispersion of the host galaxy

The host galaxy of AT2020ocn was observed by SDSS on 12 February 2008, that is, about 12 years before the flare occurred. No narrow emission lines are visible in the spectrum, indicating that the host is a quiescent galaxy. We divide the flux-calibrated spectrum by the median flux value to quasi-normalize the spectrum. Then, we use the penalized pixel fitting routine^43^ combined with the MILES single stellar population template library^44^ to measure the velocity dispersion of the stellar absorption lines (σ_*_). We conservatively mask the locations of prominent emission lines during this process (although none are apparent). Following ref. ^45^, we resample the spectrum within the errors and repeat the fitting procedure 1,000 times and take the mean and standard deviation as the velocity dispersion σ_*_ and its uncertainty. We find σ_*_ = 82 ± 4 km s^−1^, which translates into a black hole mass of log_10_(*M*/*M*_⊙_) = 6.4 ± 0.6 using the *M*–σ_*_ relation of ref. ^46^. Here we have added the measurement uncertainty in quadrature with the systematic uncertainty in the *M*–σ_*_ relation.

### Estimating the statistical significance of the 15-day X-ray flux modulations

We use the following procedure to estimate the global statistical significance of the 15-day quasi-periodicity seen in the X-ray light curve in Fig. 1a. The main steps are as follows:

1. estimating the nature of the continuum noise in the periodogram;
2. simulating a large number of light curves that follow the above continuum variability;
3. sampling these simulated light curves exactly as the real and observed data; and
4. computing the LSPs exactly as done on real data and from these estimating the likelihood of finding a QPO as strong as the one found in real data with a wide range of coherence values.

We describe each of these steps in detail below.

#### Estimating the nature of the continuum in the periodogram

A quick visual inspection of the X-ray light curve (Fig. 1a) suggests that there are seven to eight prominent flares roughly 15 days apart (see vertical dashed lines in Fig. 1a to guide the eye). The second peak appears to be blended with the third one. These regular flares terminate beyond MJD 59171 as a corona is eventually formed, and these observations are discussed in a separate paper. We started our timing analysis by computing the LSP of the observed, background-subtracted 0.3–1.0 keV X-ray light curve using all the data until MJD 59171. The LSP was computed exactly as described in ref. ^7^ and normalized as in ref. ^8^. As expected, there is a broad peak in the LSP around 15 days. There are also broad peaks in the LSP at integer harmonics, that is, 2 × 15 days, 4 × 15 days, and smaller peaks near $\frac{1}{2}\times 15\,{\rm{days}}$ and $\frac{1}{4}\times 15\,{\rm{days}}$ (Fig. 2). To assess the nature of the noise, that is, the distribution of the power values within the LSP, we used the Kolmogorov–Smirnov and Anderson–Darling tests for white noise. To assess the nature of the LSP in the vicinity of about 15 days, we used the power values that correspond to timescales longer than 3 days and excluded bins near 15 days, 2 × 15 days and 4 × 15 days. Following the procedure outlined in section 2.2.2 of ref. ^35^, both the Kolmogorov–Smirnov and Anderson–Darling tests suggest that the null hypothesis that these LSP powers (normalized by mean) below 3 days are white cannot be rejected at even the 90% confidence level (Extended Data Fig. 3).

The average of the power values corresponding to timescales longer than 3 days (excluding those near 15 days, 2 × 15 days and 4 × 15 days) is elevated compared with the average value for shorter than 3 days. This could be because of two reasons: (1) the contribution from the wings of the broad peaks at 15 days and its harmonics; or (2) genuine red noise. Therefore, we compute the FAPs separately for both these cases.

Red noise is a common type of variability noise that is predicted from both general relativistic magnetohydrodynamic simulations (see, for example, ref. ^47^) and X-ray observations and is, in general, described analytically by a power law or a bending power law^48^. We rebinned the LSP by a factor of 10 and fitted it with a power-law + constant and a bending power-law + constant models. The former and the latter yielded a *χ*^2^/*d* of 26/48 and 24/46, respectively. For the power-law model, the best-fit index, normalization and constant values were 1.0 ± 0.3, ${1.2}_{-1.2}^{+4.4}\times 1{0}^{-6}$ and 0.2 ± 0.1, respectively. For the bending power-law model, the best-fit normalization, low-frequency power-law index, bend frequency, high-frequency index and constant values were 0.7 ± 5.0, 0.1 ± 0.5, (2.5 ± 0.3) × 10^−6^ Hz, 15 ± 13 and 0.38 ± 0.03, respectively. Below, we describe our analysis for the power-law + constant models, but we repeat the same procedure for the bending power-law case.

It is known that the best-fit power-law index value inferred from modelling the LSP can be biased if the time series is unevenly sampled^49^. To test whether the current sampling could have biased our estimate of the index, we carried out the following tests.

First, using the algorithm of ref. ^50^, we simulated 10,000 time series, the power spectrum of which is defined by the best-fit power-law + constant model of the real data, that is, an index of unity and a normalization of 1.2 × 10^−6^. This time series had a resolution of 100 s. To account for red-noise leakage^49^, we ensured each of these time series were 10 times longer than the observed baseline of about 130 days. Then, we sampled each of these 10,000 time series exactly as the observed light curve and computed their LSPs. Then we rebinned them by a factor of 10—similar to the observed LSP—and fit them with a power-law + constant model. The best-fit power-law index values had a median and standard deviation of 0.93 and 0.07, respectively (Extended Data Fig. 4), that is, consistent with the best-fit values shown in Fig. 2. This demonstrates that, for the current uneven sampling, the inferred power-law index from modelling the LSP represents the true shape of the underlying power spectrum.

#### Monte Carlo simulations of time series

Based on the analysis in the above section, we concluded that the underlying continuum can be described as white, a power-law or a bending power-law red noise. The goal in this section is to answer the following question of how often would we see a broad QPO-like feature as strong as the one seen in the observed LSP for each of these underlying noise continuum models. To address this question, we use the following methodology for six continuum models: (1) white noise; (2) best-fit power-law red-noise model corresponding to (index, normalization) = (1.0, 1.2 × 10^−6^); (3) red-noise model corresponding to (index, normalization) = (best-fit index + 1*σ* error, corresponding normalization) = (1.3, 1.0 × 10^−7^); (4) a red-noise model with (corresponding index, best-fit normalization + 1*σ* uncertainty) = (0.9, 5.6 × 10^−6^); (5) a bending-power-law red-noise model corresponding to the best-fit parameters; and (6) a bending-power-law noise corresponding to the best-fit normalization + 1*σ* uncertainty.

1. Using the algorithm of ref. ^50^, we simulated 10,000 LSPs sampled exactly as the real data and computed its median, LSP_median_.
2. We then normalized each of the 10,000 LSPs with LSP_median_.
3. We found all QPO-like features with coherence, *Q*, between 2 and 10. This was automated by carrying out a sliding window cross-correlation with a Lorentzian, the width of which at a given frequency is defined by *Q*.
4. Then we computed the sum of powers, Σ_*p*_, over the width of these features and saved the maximum of these sums, Σ_*p*,max_, that is, the strongest QPO-like feature.
5. Then we plotted a cumulative distribution function (CDF) of Σ_*p*,max_ values (Extended Data Fig. 5).
6. Finally, we ran steps 1–3 on the observed LSP to compute the Σ_*p*,max,observed_ and overlaid its position on the CDF from step 4. In all the cases, this step found the peak near 15 days.

Based on this analysis, we concluded that the QPO feature near 15 days is statistically acceptable (Extended Data Fig. 5).

### X-ray spectra analysis

#### Preliminary X-ray spectral modelling with XMM-Newton/EPIC-pn data

**Extended Data Fig. 6:**
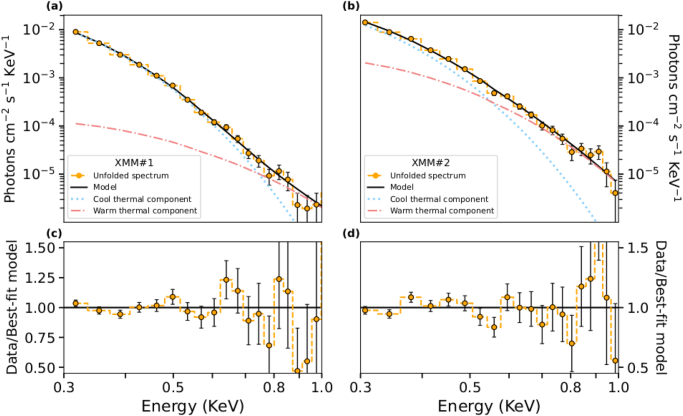
AT2020ocn’s *XMM-Newton* X-ray spectra. The best-fit model consists of two thermal components. These spectra are available as supplementary files. All the errorbars represent 90% uncertainties.

**Extended Data Fig. 7:**
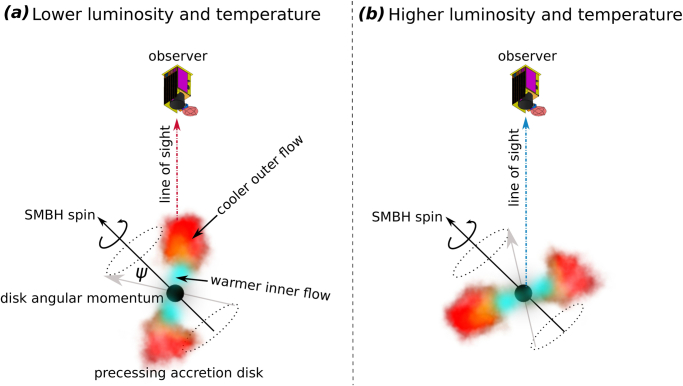
A simplified schematic of a potential model showing Lense-Thirring precession of an inner disk. In the left precession phase a), view of the inner/warm disk is obstructed and, consequently, we would see lower luminosity and temperature. In the right panel b), the inner/warm disk is visible which leads to higher observed temperature and luminosity. Relative sizes are not to scale.

We started our spectral modelling with XMM#2, which had 3,214 counts in the 0.3–1.2 keV band. Because the spectrum was soft, we first fit it with a single thermal component, tbabs*ztbabs*zashift*diskbb in XSPEC leaving all but tbabs parameters to be free to vary. The Milky Way column density was fixed at 1.3 × 10^20^ cm^−2^ using the HEASARC tools of NASA (https://heasarc.gsfc.nasa.gov/cgi-bin/Tools/w3nh/w3nh.pl). This gave a poor fit with C-statistic/degrees of freedom (dof) of 44.0/19. Strong systematic residuals above 0.6 keV were evident. Next, we added a power-law component that yielded a better C-stat/dof of 25.8/17. However, the best-fit power-law index was extreme with a value of ${5.7}_{-2.1}^{+1.2}$. For comparison, typical index values for persistently accreting SMBHs, that is, AGN, are below 2 with a value near 3 considered to be extreme^51^. Index values around 6 are unphysical because they imply an unrealistically high intrinsic luminosity when extrapolated to lower energies. These steep index values can be explained by the fact that in the narrow bandpass of 0.3–1.2 keV we are fitting the Wien’s portion of a thermal component, which naturally leads to a steep index when modelled with a power law. Therefore, we next tried fitting two thermal components, that is, tbabs*ztbabs*zashift(diskbb+diskbb) in XSPEC. This resulted in a C-statistic/dof of 22.9/17. The two temperatures were ${0.064}_{-0.010}^{+0.008}\,{\rm{keV}}$ and ${0.119}_{-0.027}^{+0.068}\,{\rm{keV}}$ (Extended Data Fig. 6). We also tried the tbabs*ztbabs*zashift(diskbb+blackbody) model, which resulted in a similar C-statistic/dof value of 22.8/17. In all these cases, the fit required the neutral column density of the host, that is, ztbabs, to be 0. We repeated the same analysis on a few of the NICER spectra from near the peaks of the flares in Fig. 1a and concluded that two thermal components describe the data better than a single thermal component. The exact choice of the thermal components, that is, diskbb versus blackbody, does not matter for the overall conclusions.

#### Time-resolved X-ray spectral modelling using NICER data

Motivated by the above spectral modelling and recent detections of two temperature X-ray spectra^52,53^, we adopted a model with two thermal components. To track the two components over approximately the first 4 months, we extracted composite NICER spectra by combining the neighbouring GTIs to have at least 1,000 counts in each spectra in the 0.3–1.0 keV band and more than 50 counts in the 0.75–1.0 keV band. This resulted in 165 spectra between MJDs 59041 and 59171 with median (standard deviation) counts of 3,700 (2,400). These were fit separately with a single disk blackbody tbabs*zashift*(diskbb) in XSPEC and two disk blackbodies tbabs*zashift(diskbb+diskbb). In all the cases, the column density near the host (ztbabs) was pegged by the fit to 0. For each spectrum, we computed the evidence ratio as per the Akaike information criterion for two disks with respect to a single disk model. If the evidence ratio was less than 10, we fixed the cool disk to a value of 0.062 keV and estimated the luminosity of the cool disk. By contrast, if the evidence ratio was greater than 10, the temperature of the cool thermal component was allowed to be free (Fig. 3).

#### X-ray flux variations are not driven by neutral column density changes near the TDE

To test whether the observed changes in X-rays are driven by changes in column density, we extracted higher count NICER spectra from near the peaks of the early time flares and compared them with the spectra between the flares by letting the neutral column density be a free parameter. In all these cases, the best-fit column density of the host was again close to 0. For instance, a composite NICER spectrum using the data taken between MJDs 59073.54 and 59074.45 had roughly 32,200 counts in the 0.3–1.0 keV band. Modelling this spectrum with tbabs*ztbabs*zashift*(diskbb+diskbb) yielded a best-fit column of the ztbabs component to be close to 0. For comparison, the 0.3–1.0 keV flux during this time was about 3 × 10^−12^ erg s^−1^ cm^−2^, which is a factor of 6 and 4 higher compared with XMM#1 and XMM#2 epochs. Another example is a spectrum obtained using the data between MJDs 59087.085 and 59087.877 that had about 21,650 counts. The source flux during this epoch was 3.5 × 10^−12^ erg s^−1^ cm^−2^ and again the best-fit ztbabs column was close to 0. Based on these tests, we concluded that the observed changes in the X-ray flux were not driven by changing the neutral column near the TDE.

### Spin estimate assuming rigid body precession

If rigid body disk precession is driving these 15-day modulations, we can use the observed period to constrain the disrupting the spin of SMBH^1^. We calculated the precession period following ref. ^1^, taking the power law of the surface density profile to be *s* = −3/2, which corresponds to the radiation-pressure-dominated inner region of the standard disk model^54,55^. In this case, the timescale is principally determined in the outer disk regions, and we find that *t*_p_ = (π/*a*)(*R*_out_/*R*_g_)^3^(*GM*/*c*^3^) × *ξ*(*R*_in_/*R*_out_), where *a* is the dimensionless black hole spin, *R*_g_ = *GM*/*c*^2^ is the gravitational radius of the black hole, and *ξ*(*R*_in_/*R*_out_) is a dimensionless function that is weakly dependent on the ratio of the inner and outer disk radii (for *R*_in_/*R*_out_ → 0, we have *ξ* → 1/4). *G* and *c* are the gravitational constant and the speed of light, respectively. Using this approximation, taking *R*_out_ to be the circularization radius of the debris, and inverting the equation for the precession timescale, we can calculate the black hole spin as

1 $$a\approx 0.1{\left(\frac{{t}_{{\rm{p}}}}{15.9{\rm{d}}{\rm{a}}{\rm{y}}{\rm{s}}}\right)}^{-1}\,{(\beta /2)}^{-3}\,(\xi /0.5)\,{M}_{\star ,\odot }^{-1}\,{R}_{\star ,\odot }^{3}{M}_{7}^{-1},$$

where *β* = *R*_t_/*R*_p_ ≃ 2 is the impact parameter of the stellar orbit required to just fully disrupt a solar-like star^26^, *M*_⋆,⊙_ and *R*_⋆,⊙_ are the mass and radius of the star in solar units, and *M*_7_ is the SMBH mass scaled by 10^7^*M*_⊙_.

From this equation, we can see that to accurately constrain the spin with this model, we require accurate constraints on the parameters *β*, *M*_⋆,⊙_, *R*_⋆,⊙_ and *M*. However, if we take standard TDE parameters (as above), and an SMBH mass estimate of 10^6.4^*M*_⊙_, then we find that *a* ≈ 0.15 is required to generate the 15-day period. We also explored varying the power-law index, *s*, of the surface density profile in the range −3/2 to 3/4 and found that for SMBH masses about 10^7^*M*_⊙_ the value of *s* makes little difference to this estimate, whereas at the lower end of the black hole mass range, the value of *s* can make a significant difference with *s* = −3/2 providing the largest spin estimate. Thus, considering a range of possible models, we conclude that $0.05\lesssim \left|a\right|\lesssim 0.5$ (Fig. 4). Future modelling of the multi-wavelength emission of AT2020ocn, especially the high-cadence X-ray observations with numerical simulations, will provide a more detailed understanding of the accretion flow structure and even tighter constraints on the SMBH spin.

### Lack of similar signals in literature

There are three factors that dictate the possibility of detecting disk precession in TDEs: (1) the time for stellar debris to circularize and form a disk after disruption; (2) the time for the disk to become thin and align with the black hole spin (see, for example, figure 2 of ref. ^1^); and (3) the geometric orientation of the system with respect to our line of sight so that the flux density variations are maximized. The circularization timescale marks the beginning of disk precession, whereas the disk alignment time marks its termination. Even if high-cadence observations are made between the above two epochs, a system that is close to face-on will not show any detectable X-ray modulations. Thus, the search for Lense–Thirring precession in TDEs is best suited for close to edge-on systems that promptly form an accretion disk, that is, systems that show early X-ray emission. Only a handful of TDEs have been monitored with high-cadence in the X-rays, and this in combination with the geometric constraints might explain the lack of such signatures in the previously known TDEs.

### Discussion of various models

#### Partial TDE

The lack of emission before the initial detection implies that if the X-ray modulations are generated by a repeating partial TDE, the star must have been placed on a 15-day orbit through some mechanism. Tidal interactions alone cannot dissipate enough energy to yield the required orbit^10^, and for the black hole mass inferred for AT2020ocn Hills capture would require an approximately 1,000-s orbit of the original binary and a sub-solar-radius separation^10^ for solar-like (that is, one solar mass) binary components. Furthermore, the fact that the optical and UV lightcurves show evolution on about 30−50 day timescales implies that the fallback time is of this order, which would be longer than the 15-day orbit of the star (which is presumably responsible for modulating the X-ray emission). It seems unreasonable for the fallback time to be longer than the orbital time of the star, rendering this scenario extremely unlikely.

#### Variability from discrete stream collisions

We can expect that some variability in the TDE accretion process comes from the dynamics of the returning debris stream. For example, the recurrent X-ray flares could, in principle, result from the infall of material onto the black hole from self-intersection shocks. In particular, recent simulations^12,56^ have shown that the self-intersection of the debris stream from a deep TDE leads to a geometrically inflated, slowly evolving, quasi-spherical flow at large radii that can extend to hundreds to thousands of gravitational radii. However, on small scales and very near the horizon of the black hole, where the around keV emission would originate^12^, we found that the flow and the accretion is modulated on timescales comparable to the freefall time from the self-intersection shock, as material dissipates energy and periodically falls to the black hole on this timescale. Geometrically it follows that the self-intersection radius *r*_SI_—assuming that the self-intersection arises from the general relativistic advance of periapsis—is related to the pericentre distance of the star *r*_p_ using

2 $${r}_{{\rm{SI}}}=\frac{{r}_{{\rm{p}}}^{2}}{3\pi GM/{c}^{2}},$$

where *M* is the mass of the black hole. If we associate 15 days with the freefall time *t*_ff_ from the self-intersection shock, then ${r}_{{\rm{SI}}}\simeq {(\sqrt{GM}{t}_{{\rm{ff}}})}^{2/3}$, and letting *M* = 10^6^*M*_⊙_, equation (2) yields a pericentre distance of *r*_p_ ≈ 200*GM*/*c*^2^. This distance is a factor of a few larger than the classical tidal disruption radius of a solar-like star by a 10^6^*M*_⊙_ black hole, and the self-intersection radius is *r*_SI_ ≈ 4,400*GM*/*c*^2^, that is, highly spatially extended compared with a geometrically thin disk at the tidal radius.

In this scenario, the material from the self-intersection shock would fall towards the black hole to form a small-scale disk. This disk could generate the higher temperature X-ray emission (that is, the warmer component shown by the X-ray analysis of AT2020ocn). The higher disk temperature, and thus the additional pressure support, could inflate the disk to the point that it once again obscures our line of sight, resulting in the rapid shutoff of the X-ray emission. However, to make the above estimate of the pericentre distance that is required to generate the 15-day timescale commensurate with the tidal radius of a solar-like star requires the black hole mass to be around 10^5^*M*_⊙_, which is smaller than that implied by the M–σ_*_ scaling for AT2020ocn.

Another possibility is the variability induced by the stream colliding with the accretion disk. In the case that the disk is misaligned to the black hole spin and precessing, the radius at which the stream hits the disk varies with time. This, in turn, affects the accretion rate through the disk as the angle between the stream and disk angular momentum varies. This affect arises both through the radius at which the mass is added to the disk, and through the amount of angular momentum cancellation that is caused by the addition of material with a roughly constant angular momentum direction to a disk with a time-varying angular momentum direction. As noted in the main text, these complications could plausibly explain the more erratic behaviour of the X-ray lightcurve than can be explained by a precessing, planar disk, but we leave a detailed investigation of these features to future work.

#### Radiation-pressure instability

Inner portions of standard thin disks are unstable when dominated by radiation pressure^3^. This can eventually lead to quasi-periodic flares in the accretion rate explaining the behaviour of some changing-look AGN. The model proposed in ref. ^57^ consists of an inner advection-dominated hot flow, which is a stable optically thin solution, and the outer standard thin disk, whose inner zone with the width of Δ*R* is radiation-pressure dominated and located on an unstable branch in the accretion rate–surface density plane. The flares due to radiation-pressure instability recur on the timescale, *τ*_flare_, shorter than the standard viscous timescale at the distance *R*, that is, *τ*_flare_ = *τ*_visc_Δ*R*/*R*, where *τ*_visc_ is the viscous timescale that depends on the distance *R*, the viscosity *α* parameter, and the flow scale-height *H*. The unstable zone forms for a relative (Eddington) accretion rate larger than $\dot{m}\gtrsim 0.15{(\alpha /0.1)}^{41/29}{(M/1{0}^{6}{M}_{\odot })}^{-1/29}$ (ref. ^57^), which can be achieved in AT2020ocn for *M* ≲ 10^6.7^*M*_⊙_.

As was shown in ref. ^58^, using global time-dependent calculations without the assumption of the hot inner advection-dominated accretion flow, the model can be fine-tuned enough, including corona and sufficiently strong magnetic field, to produce quasi-periodic flux changes by an order of magnitude on the timescale as short as about 10 days, when the disk is small enough (similar to that for TDEs) or interrupted by a gap. For the early phases of the TDE, we expect Eddington to super-Eddington accretion rates, and in that case, the accretion disk is expected to be a geometrically thicker slim disk that is stabilized by advection. The radiation-pressure instability model is still applicable for a standard disk, and the advective terms are included in the calculations using radial derivatives of density and temperature (time-dependent numerical simulations tailored to AT2020ocn will be presented in a separate study). For AT2020ocn, this is relevant if its SMBH is heavier, for example, for *M* ≈ 10^7^*M*_⊙_, that is, if the Eddington ratio is $\dot{m}\approx 0.1$, so that the accretion disk can be of a standard type. The model is applicable as well for higher accretion rates. However, a challenge to the instability model is the fact that the flares of AT2020ocn weaken after a few months (Fig. 1a). This can happen if the magnetic field gets sufficiently strong, for example, owing to the dynamo effect, which tends to stabilize the disk^59^. In that case, we would expect the amplitude to gradually decrease with the magnetic field build-up.

#### Disk tearing

In the main text, we discussed the response of the TDE disk to Lense–Thirring precession in the case that the disk is sufficiently hot so that warp waves, which propagate at a velocity of roughly half the sound speed, are able to communicate the precession efficiently to create rigid precession of the (warped) disk. If the disk is thin enough such that it develops a strong warp, then it can be unstable to disk tearing, in which the disk breaks apart into discrete rings that precess at roughly the local Lense–Thirring rate^23,60^. The disk-tearing instability is understood from an analytical standpoint^61–63^, and the associated nonlinear behaviour has now been explored in a variety of numerical simulations, including Lagrangian and Eulerian codes; for example, refs. ^23,64^ and in both the high- and low-viscosity regimes^65^. Recently, a protoplanetary disk in the multi-stellar system GW Ori has been seen to harbour misaligned and broken rings of gas using spatially resolved observations, and this has been ascribed to the disk-tearing process^66^ (in which the precession was driven by the gravitational torque from the orbiting stars; ref. ^67^). The variability induced by the disk-tearing process can be because of both geometric effects (for example, rings precessing through the line of sight) or intrinsic disk variability (for example, enhancement of the central accretion rate due to rapid accretion from interacting rings). The timescale for the variability is of the order of the local Lense–Thirring precession timescale in the unstable region of the disk^20^. For the timescale to be about 15.9 days, this would suggest rings precessing at radii of ${R}_{{\rm{prec}}}\approx 35\,{R}_{{\rm{g}}}\,{(a/0.5)}^{1/3}{M}_{6}^{-1/3}$, where *M*_6_ = *M*/10^6^ *M*_⊙_. Using the simple estimate provided by ref. ^23^ for which we would expect the disk to break into discrete rings, we have *R*_break_ ≈ 15*R*_g_ (*a*/0.5)^2/3^(*α*/0.1)^−2/3^(*H*/*R*/0.1)^−2/3^, where *α* ≈ 0.1 (ref. ^21^) is the Shakura–Sunyaev disk viscosity parameter and *H*/*R* is the disk angular semi-thickness. It is, therefore, possible to find reasonable parameters that are consistent with what we know about AT2020ocn to bring these into agreement. For example, with a black hole mass of 10^6.4^*M*_⊙_, a spin of *a* ≈ 0.5, *α* = 0.1 and *H*/*R* ≈ 0.05 (consistent with, for example, the disk model of ref. ^68^ with $\dot{M}/{\dot{M}}_{{\rm{Edd}}}=0.3$), we can expect disk tearing to occur at a radius of about 25*R*_g_ with a period of around 15 days. Thus, it is possible that the variability observed in the X-ray lightcurve from AT2020ocn is because of either a Lense–Thirring precession of a rigidly precessing and radially extended portion of the disk (as discussed in the main text) or a Lense–Thirring precession of an unstable region of the disk that has broken into discrete rings.

## Online content

Any methods, additional references, Nature Portfolio reporting summaries, source data, extended data, supplementary information, acknowledgements, peer review information; details of author contributions and competing interests; and statements of data and code availability are available at 10.1038/s41586-024-07433-w.

### Supplementary information

Peer Review File

## Figures and Tables

**Extended Data Table 1:**
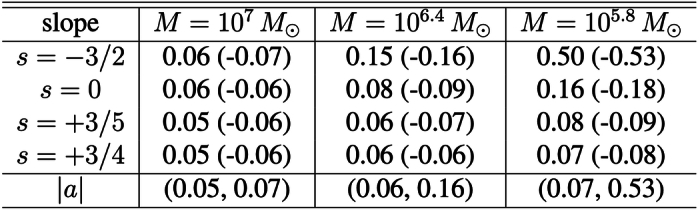
Summary of spin constraints for AT2020ocn SMBH for different surface-density slopes of the precessing accretion flow and the range of SMBH masses

The negative spin values in parentheses correspond to the retrograde spin. The outer radius of the precessing disk is fixed at the corresponding tidal radius for a Sun-like star. For the radial surface-density profile, we use the prescription Σ ∝ *R*^−*s*^. The last line summarizes the spin interval for a given SMBH mass.

## Data Availability

All the NICER, XMM-Newton and Swift data presented here are publicly available and can be found in the NASA archives at https://heasarc.gsfc.nasa.gov/cgi-bin/W3Browse/w3browse.pl. Data shown in Figs. 1 and 3 can be found on Zenodo 10.5281/zenodo.10062825 (ref. ^69^). The XMM-Newton spectra are available at 10.5281/zenodo.8252931 (ref. ^70^). Time-resolved NICER spectra can be downloaded from 10.5281/zenodo.8253537 (ref. ^71^).

## Code availability

A sample Python code necessary to read and plot the data can be found on Zenodo at 10.5281/zenodo.10062825 (ref. ^69^).

## References

[1] Stone N, Loeb A (2012). Observing Lense–Thirring precession in tidal disruption flares. Phys. Rev. Lett..

[2] Franchini A, Lodato G, Facchini S (2016). Lense–Thirring precession around supermassive black holes during tidal disruption events. Mon. Not. R. Astron. Soc..

[3] Lightman AP, Eardley DM (1974). Black holes in binary systems: instability of disk accretion. Astrophys. J. Lett..

[4] Janiuk A, Czerny B, Siemiginowska A (2000). Radiation pressure instability as a variability mechanism in the microquasar GRS 1915+105. Astrophys. J. Lett..

[5] Gezari S (2020). AT2020ocn/ZTF18aakelin: tidal disruption event that is brightening in the X-rays. The Astronomer’s Telegram.

[6] Hammerstein E (2023). The final season reimagined: 30 tidal disruption events from the ZTF-I survey. Astrophys. J..

[7] Scargle JD (1982). Studies in astronomical time series analysis. II. Statistical aspects of spectral analysis of unevenly spaced data. Astrophys. J..

[8] Horne JH, Baliunas SL (1986). A prescription for period analysis of unevenly sampled time series. Astrophys. J..

[9] McClintock, J. E. & Remillard, R. A. in *Compact Stellar X-ray Sources* Vol. 39 (eds Lewin, W. et al.) 157–213 (Cambridge Univ. Press, 2006).

[10] Cufari M, Coughlin ER, Nixon CJ (2022). Using the Hills mechanism to generate repeating partial tidal disruption events and ASASSN-14ko. Astrophys. J. Lett..

[11] Bonnerot C, Lu W (2020). Simulating disc formation in tidal disruption events. Mon. Not. R. Astron. Soc..

[12] Andalman ZL, Liska MTP, Tchekhovskoy A, Coughlin ER, Stone N (2022). Tidal disruption discs formed and fed by stream-stream and stream-disc interactions in global GRHD simulations. Mon. Not. R. Astron. Soc..

[13] Ingram A, Done C (2010). A physical interpretation of the variability power spectral components in accreting neutron stars. Mon. Not. R. Astron. Soc..

[14] Motta SE, Belloni TM, Stella L, Muñoz-Darias T, Fender R (2014). Precise mass and spin measurements for a stellar-mass black hole through X-ray timing: the case of GRO J1655-40. Mon. Not. R. Astron. Soc..

[15] Nixon C, Salvesen G (2014). A physical model for state transitions in black hole X-ray binaries. Mon. Not. R. Astron. Soc..

[16] Belloni T, Méndez M, King AR, van der Klis M, van Paradijs J (1997). An Unstable Central Disk in the Superluminal Black Hole X-Ray Binary GRS 1915+105. Astrophys. J. Lett..

[17] Muno MP, Morgan EH, Remillard RA (1999). Quasi-periodic oscillations and spectral states in GRS 1915+105. Astrophys. J..

[18] Altamirano D (2011). The faint “heartbeats” of IGR J17091-3624: an exceptional black hole candidate. Astrophys. J. Lett..

[19] Belloni T, Méndez M, King AR, van der Klis M, van Paradijs J (1997). A unified model for the spectral variability in GRS 1915+105. Astrophys. J. Lett..

[20] Raj A, Nixon CJ (2021). Disk tearing: implications for black hole accretion and AGN variability. Astrophys. J..

[21] Martin RG, Nixon CJ, Pringle JE, Livio M (2019). On the physical nature of accretion disc viscosity. New Astron..

[22] Papaloizou JCB, Pringle JE (1983). The time-dependence of non-planar accretion discs. Mon. Not. R. Astron. Soc..

[23] Nixon C, King A, Price D, Frank J (2012). Tearing up the disk: how black holes accrete. Astrophys. J. Lett..

[24] Wu S, Coughlin ER, Nixon C (2018). Super-Eddington accretion in tidal disruption events: the impactof realistic fallback rates on accretion rates. Mon. Not. R. Astron. Soc..

[25] Ulmer A (1999). Flares from the tidal disruption of stars by massive black holes. Astrophys. J..

[26] Guillochon J, Ramirez-Ruiz E (2013). Hydrodynamical simulations to determine the feeding rate of black holes by the tidal disruption of stars: the importance of the impact parameter and stellar structure. Astrophys. J..

[27] Roth N, Kasen D, Guillochon J, Ramirez-Ruiz E (2016). The X-Ray through optical fluxes and line strengths of tidal disruption events. Astrophys. J..

[28] Auchettl K, Guillochon J, Ramirez-Ruiz E (2017). New physical insights about tidal disruption events from a comprehensive observational inventory at X-ray wavelengths. Astrophys. J..

[29] Guolo, M. et al. A systematic analysis of the X-ray emission in optically selected tidal disruption events: observational evidence for the unification of the optically and X-ray selected populations. Preprint at arxiv.org/abs/2308.13019 (2023).

[30] Bricman K, Gomboc A (2020). The prospects of observing tidal disruption events with the Large Synoptic Survey Telescope. Astrophys. J..

[31] Bellm EC (2019). The Zwicky Transient Facility: system overview, performance, and first results. Publ. Astron. Soc. Pac..

[32] Planck Collaboration. (2020). Planck 2018 results. VI. Cosmological parameters. Astron. Astrophys..

[33] Wright EL (2006). A cosmology calculator for the world wide web. Publ. Astron. Soc. Pac..

[34] Pasham DR (2023). The birth of a relativistic jet following the disruption of a star by a cosmological black hole. Nat. Astron..

[35] Pasham DR (2021). Evidence for a compact object in the aftermath of the extragalactic transient AT2018cow. Nat. Astron..

[36] Remillard RA (2022). An empirical background model for the NICER X-ray timing instrument. Astron. J..

[37] Kaastra JS, Bleeker JAM (2016). Optimal binning of X-ray spectra and response matrix design. Astron. Astrophys..

[38] Arnaud, K. A. in *Astronomical Data Analysis Software and Systems V* Vol. 101 (eds Jacoby, G. H. & Barnes, J.) 17–20 (Astrophysics Society of the Pacific, 1996).

[39] Frederick, S. *ZTF/iPTF Transient Discovery Report for 2020-07-07*. Report No. 2020-2059 (Transient Name Server, 2020).

[40] Masci FJ (2019). The Zwicky Transient Facility: data processing, products, and archive. Publ. Astron. Soc. Pac..

[41] Schlafly EF, Finkbeiner DP (2011). Measuring reddening with Sloan Digital Sky Survey stellar spectra and recalibrating SFD. Astrophys. J..

[42] Roming PWA (2005). The *Swift* Ultra-Violet/Optical Telescope. Space Sci. Rev..

[43] Cappellari M (2017). Improving the full spectrum fitting method: accurate convolution with Gauss-Hermite functions. Mon. Not. R. Astron. Soc..

[44] Falcón-Barroso J (2011). An updated MILES stellar library and stellar population models. Astron. Astrophys..

[45] Wevers T (2017). Black hole masses of tidal disruption event host galaxies. Mon. Not. R. Astron. Soc..

[46] McConnell NJ, Ma C-P (2013). Revisiting the scaling relations of black hole masses and host galaxy properties. Astrophys. J..

[47] Mishra B, Kluźniak W, Fragile PC (2020). Relativistic, axisymmetric, viscous, radiation hydrodynamic simulations of geometrically thin discs. II. Disc variability. Mon. Not. R. Astron. Soc..

[48] Markowitz A (2003). X-ray fluctuation power spectral densities of Seyfert 1 galaxies. Astrophys. J..

[49] Uttley P, McHardy IM, Papadakis IE (2002). Measuring the broad-band power spectra of active galactic nuclei with *RXTE*. Mon. Not. R. Astron. Soc..

[50] Timmer J, Koenig M (1995). On generating power law noise. Astron. Astrophys..

[51] Dadina M (2008). Seyfert galaxies in the local Universe (*z* ≤ 0.1): the average X-ray spectrum as seen by *BeppoSAX*. Astron. Astrophys..

[52] Miniutti G (2019). Nine-hour X-ray quasi-periodic eruptions from a low-mass black hole galactic nucleus. Nature.

[53] Liu X-L, Dou L-M, Chen J-H, Shen R-F (2022). The UV/Optical peak and X-ray brightening in TDE candidate AT 2019azh: a case of stream-stream collision and delayed accretion. Astrophys. J..

[54] Shakura NI, Sunyaev RA (1973). Black holes in binary systems. Observational appearance. Astron. Astrophys..

[55] Novikov, I. D. & Thorne, K. S. in *Black Holes, Les Astres Occulus* 343–450 (Gordon and Breach, 1973).

[56] Sądowski A, Tejeda E, Gafton E, Rosswog S, Abarca D (2016). Magnetohydrodynamical simulations of a deep tidal disruption in general relativity. Mon. Not. R. Astron. Soc..

[57] Sniegowska M, Czerny B, Bon E, Bon N (2020). Possible mechanism for multiple changing-look phenomena in active galactic nuclei. Astron. Astrophys..

[58] Śniegowska M, Grzȩdzielski M, Czerny B, Janiuk A (2023). Modified models of radiation pressure instability applied to 10, 10^5^, and 10^7^
*M*_⊙_ accreting black holes. Astron. Astrophys..

[59] Kaur K, Stone NC, Gilbaum S (2023). Magnetically dominated discs in tidal disruption events and quasi-periodic eruptions. Mon. Not. R. Astron. Soc..

[60] Raj A, Nixon CJ, Doğan S (2021). Disk tearing: numerical investigation of warped disk instability. Astrophys. J..

[61] Ogilvie GI (2000). An alpha theory of time-dependent warped accretion discs. Mon. Not. R. Astron. Soc..

[62] Doğan S, Nixon CJ, King AR, Pringle JE (2018). Instability of warped discs. Mon. Not. R. Astron. Soc..

[63] Doğan S, Nixon CJ (2020). Instability of non-Keplerian warped discs. Mon. Not. R. Astron. Soc..

[64] Liska M (2021). Disc tearing and Bardeen–Petterson alignment in GRMHD simulations of highly tilted thin accretion discs. Mon. Not. R. Astron. Soc..

[65] Drewes NC, Nixon CJ (2021). On the dynamics of low-viscosity warped disks around black holes. Astrophys. J..

[66] Kraus S (2020). A triple-star system with a misaligned and warped circumstellar disk shaped by disk tearing. Science.

[67] Nixon C, King A, Price D (2013). Tearing up the disc: misaligned accretion on to a binary. Mon. Not. R. Astron. Soc..

[68] Strubbe LE, Quataert E (2009). Optical flares from the tidal disruption of stars by massive black holes. Mon. Not. R. Astron. Soc..

[69] Pasham, D. Data to reproduce Figs 1 and 3 of paper titled “Lense-Thirring Precession after a Supermassive Black Hole Disrupts a Star”. *Zenodo*10.5281/zenodo.10062825 (2023). 10.1038/s41586-024-07433-w PMC11168925 38778113

[70] Pasham, D. XMM spectra of AT2020ocn. *Zenodo*10.5281/zenodo.8252931 (2023).

[71] Pasham, D. NICER spectra of AT2020ocn. *Zenodo*10.5281/zenodo.8253537 (2024).

