# Supplementary material for: Lense–Thirring precession after a supermassive black hole disrupts a star
Source: Nature. 2024 May 22;630(8016):325–8. doi: 10.1038/s41586-024-07433-w (PMC11168925; doi:10.1038/s41586-024-07433-w)
Supplement: Supplementary file 1 — Peer Review File [file 41586_2024_7433_MOESM1_ESM.pdf]

Peer Review File

Manuscript Title: Lense-Thirring Precession after a Supermassive Black Hole Disrupts a Star

Reviewer Comments & Author Rebuttals

Reviewer Reports on the Initial Version:

Referees' comments:

Referee #1 (Remarks to the Author):

I read with interest the manuscript entitled "Evidence for Lense-Thirring precession of a newly formed disk around a supermassive black hole after it disrupts a star" by Pasham et al. This paper presents an intriguing observation, which could potentially be the first observational evidence of Lense-Thirring (LT) precession following a tidal disruption event. The remarkable quality of the X-ray light curve enables the authors to perform a meticulous timing analysis, which reveals a prominent peak in the Lomb-Scargle periodogram (LSP) occurring at a period of approximately 15 days.

On the methodology aspect, I found the timing analysis very convincing, even though I was, at first, puzzled by the apparent absence of the second peak in the light curve. Although I understand that many physical processes may be at play in this light curve, I would recommend discussing why this peak could be missing in the LT precession scenario (see my other comments below). The authors conservatively tested several noise models to evaluate the significance of the detection, even though they demonstrated that the simplest white noise model cannot be ruled out. All noise models tested seem incapable of producing a peak in the LSP as high as observed in the real data, confirming the high significance of the detection. I think this provides sufficient evidence to claim the detection of Quasi-Periodic Oscillations (QPO) in this light curve.

The authors then discuss the physical process at the origin of this periodic signal, which they attribute to the precession of the newly formed accretion disk. This interpretation is not unequivocal, but this is clearly stated in the abstract (and alternative scenarios are discussed in detail in the Supplementary Materials). After reading the argumentation developed in the paper, I agree that the main hypothesis, i.e., an accretion disk in LT precession, is indeed the most likely explanation for the observed data.

Overall, I found the manuscript very clear, and I think it would be of interest to a large audience. I only have a few comments and suggestions that I am listing below:

In the Abstract, you quote a period of 17.0 +1.2/-2.4 days. I can’t trace where this value and the uncertainties have been computed. Are they coming from a fit in direct space or are they derived from the LSP? Can you give more details on how this value and the uncertainties have been computed in the methodology section? It seems that a 17-day period provides a significantly worse fit to the data than 15 days.

In line 38, the authors report a statistical significance > 3.9 sigma. It is not clear to which of the 6

noise models of EDF 6 this value corresponds. From the plots, I guess this corresponds to model d) (Powerlaw red noise with Norm_best + 1sigma). It would be informative to report the significance of the detection for each of the 6 noise models that have been tested (especially for the white noise model since it does not seem that there is evidence of red noise in the data).

Figure 1: Looking more closely at the data, it seems that the 2nd peak is indeed there, but it has a much lower amplitude than the other peaks. Can you comment on why this 2nd peak would be much fainter in the LT precession scenario? In Figure 2a), it seems that the temperature of the warm component also peaks a bit earlier than expected. Can you comment on that?

Figure 2: the warm thermal component periodicity seems slightly different than the one observed in the original light curve. Did you look at the LSP of the warm component temperature and luminosity? Do you also observe a significant peak at ~15 days? I would recommend providing the data for Figure 2.

Other minor comments:
L188. Mpcs --> Mpc, as I think abbreviations of units do not take plural.
L527. ADAF: The acronym is not defined.

Referee #2 (Remarks to the Author):

The result is interesting and topical.

It is somewhat confusing to see '~15 days' used throughout the main text, and also as the separation of the vertical lines in the figures, when the abstract - the first thing you read - states the quasi-period to be 17 days (albeit with large enough errors that 15 days is included). Even towards the end of the paper, the body of the paper is referring to 'the 15-day period'. This is really a stylistic / presentation issue.

I think scientifically there the questions can fall in two groups : 1. Is the quasi-period real? 2. Is the interpretation correct?

Is the signal real?

My first thought on looking at the light curve in Fig 1a is that it doesn't really (at all) look like quasi-periodically spaced flares, but rather more (to me) like the long repeating sequences of X-ray behaviour seen in the X-ray binary GRS 1915+105 and a few other similar sources (I refer the authors to Belloni et al. A&A 355, 271 [2000] and Vincentelli et al. Nature 7950, 45, [2023]). In a sense these are quasi-periodic patterns of behaviour, which would show up in a Lomb-Scargle periodogram, but they are quite different from the type-C QPOs in XRBs which are widely inferred to be associated with Lense-Thirring precession (the authors' own reference [11], Ingram & Done]. Similarly, the patterns of oscillation in GRS 1915+105 and similar sources show temperature variations as seen here.

More generally, having seen many thousands of light curves, I would not personally think you can confidently assert there is a quasi-period at all from those data, with so few cycles, half of which do not seem by eye to show a signal. That said, the authors state that it is statistically significant - I am not going to check their statistical methods, so we have to take this at face value.

So maybe there is a signal, but I don't think it looks like the type-C QPOs in XRBs which are associated with L-T precession. As a side note, I also think the Lomb-Scargle periodogram should be part of the main paper, as it is central to the result, rather than in the additional material (which people often don't read).

Is the interpretation correct?

I think L-T precession is a possible interpretation of these data, but I do think the analogy with the quasi-periodically repeating state cycles in GRS 1915+105 is more natural at face value. That a SMBH should show disc instability patterns similar to stellar-mass BH and a NS is a pretty exciting result (and for me would be as interesting as the possible L-T interpretation and rather weak spin constraint [see below]).

I have to say up front that given there are so many uncertainties I do not think the spin can really be constrained. In XRBs the frequency of the type C QPO changes dramatically and in only one case, where the full set of three frequencies predicted by the relativistic precession model were present, was a BH spin estimate made. The authors themselves note that any spin estimate depends on a number of not-very-well-known parameters, but then they simply 'take standard parameters' or use estimated quantities (it's not very clear, for example, if the estimated error in the BH mass is propagated forward to the spin estimate).

I am also curious - and I am sure this is just my ignorance - why we expect a L-T-generated QPO to dampen and disappear in TDEs as the disc aligns, whereas we don't see this in XRBs, where they persistent for millions of dynamical timescales?

Author Rebuttals to Initial Comments:

Reply to the Referee reports to Nature manuscript 2023-08-14454

"Evidence for Lense-Thirring precession of a newly formed disk around a supermassive black hole after it disrupts a star"

by Dheeraj R. Pasham, Michal Zajaček, C.J. Nixon, Eric R. Coughlin, Marzena Śniegowska, Agnieszka Janiuk, Bożena Czerny, Thomas Wevers, Muryel Guolo, Yukta Ajay, Michael Loewenstein [1]

Reviewer 1's Comments:

I read with interest the manuscript entitled "Evidence for Lense-Thirring precession of a newly formed disk around a supermassive black hole after it disrupts a star" by Pasham et al. This paper presents an intriguing observation, which could potentially be the first observational evidence of Lense-Thirring (LT) precession following a tidal disruption event. The remarkable quality of the X-ray light curve enables the authors to perform a meticulous timing analysis, which reveals a prominent peak in the Lomb-Scargle periodogram (LSP) occurring at a period of approximately 15 days.

On the methodology aspect, I found the timing analysis very convincing, even though I was, at first, puzzled by the apparent absence of the second peak in the light curve. Although I understand that many physical processes may be at play in this light curve, I would recommend discussing why this peak could be missing in the LT precession scenario (see my other comments below). The authors conservatively tested several noise models to evaluate the significance of the detection, even though they demonstrated that the simplest white noise model cannot be ruled out. All noise models tested seem incapable of producing a peak in the LSP as high as observed in the real data, confirming the high significance of the detection. I think this provides sufficient evidence to claim the detection of Quasi-Periodic Oscillations (QPO) in this light curve.

The authors then discuss the physical process at the origin of this periodic signal, which they attribute to the precession of the newly formed accretion disk. This interpretation is not unequivocal, but this is clearly stated in the abstract (and alternative scenarios are discussed in detail in the Supplementary Materials). After reading the argumentation developed in the paper, I agree that the main hypothesis, i.e., an accretion disk in LT precession, is indeed the most likely explanation for the observed data.

Overall, I found the manuscript very clear, and I think it would be of interest to a large audience. I only have a few comments and suggestions that I am listing below:

1. In the Abstract, you quote a period of 17.0 +1.2/-2.4 days. I can't trace where this value and the uncertainties have been computed. Are they coming from a fit in direct space or are they derived from the LSP? Can you give more details on how this value and the uncertainties have been computed in the methodology section? It seems that a 17-day period provides a significantly worse fit to the data than 15 days.

   Reply: The value and the uncertainties were derived from the Lomb Scargle periodogram. The value, 17 days,

[1] The answers to Referee's comments and questions are colored in blue. Changes in the revised manuscript are marked with bold face font.

corresponds to the period of the highest bin near 17 days while the errorbars correspond to the full width at half maximum. We have added this description in bold in the revised manuscript (lines 34-38 of main text).

The peak in the periodogram occurs at 17(+1.2,-2.4) but visually, the individual X-ray peaks in Fig.1a align better with 15-d vertical lines. Given the uncertainty, it is possible that the value is close to 15-d and hence we refer to this signal as the 15-d quasi-periodicity throughout the manuscript. We have added text to lines 34-38 to clarify this aspect.

2. In line 38, the authors report a statistical significance > 3.9 sigma. It is not clear to which of the 6 noise models of EDF 6 this value corresponds. From the plots, I guess this corresponds to model d) (Powerlaw red noise with Norm_best + 1sigma). It would be informative to report the significance of the detection for each of the 6 noise models that have been tested (especially for the white noise model since it does not seem that there is evidence of red noise in the data).

   Reply: We simulated 10,000 periodograms for each continuum model. A peak as strong as the one observed in the real peridogram was not seen in any of the simulated data. Thus, in each case, we can say that the false alarm probability is less than 1 in 10,000, i.e., $>$3.9 sigma. We have added text to line 41 to clarify this point.

3. Figure 1: Looking more closely at the data, it seems that the 2nd peak is indeed there, but it has a much lower amplitude than the other peaks. Can you comment on why this 2nd peak would be much fainter in the LT precession scenario? In Figure 2a), it seems that the temperature of the warm component also peaks a bit earlier than expected. Can you comment on that?

   Reply: We have added a short description about this in the main section (lines 104-108).

   Regarding the warm component peaking earlier than expected: we do not think that is statistically significant. To our eyes the second peak in kT vs time in Fig. 3a appears roughly at the same time as the peak in the middle panel of Fig. 3b.

4. Figure 2: the warm thermal component periodicity seems slightly different than the one observed in the original light curve. Did you look at the LSP of the warm component temperature and luminosity? Do you also observe a significant peak at 15 days? I would recommend providing the data for Figure 2.

   Reply: Previously, we did not look at the LSP of the warm component. However, we show it here. The strongest peak is at the same timescale of $\sim$15 days. It is noisier than than the LSP of the observed luminosity vs time because it is not sampled as well. The LSP plot is similar for warm component's luminosity. Fig. 2's data can now be accessed from zenodo. We have added the web-link to the section titled "data availability".

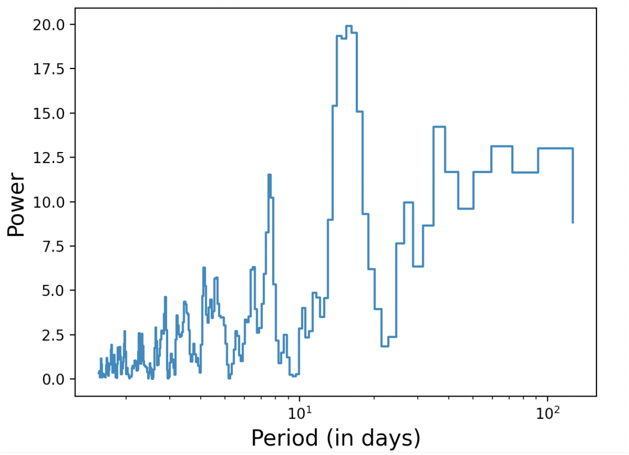

Figure 1: **Lomb Scargle periodogram of warm component's kT vs time.** The main peak is at $15.9^{+2.1}_{-2.3}$ days, consistent with the LSP peak of the observed X-ray count rate.

5. Other minor comments:

6. L188. Mpcs → Mpc, as I think abbreviations of units do not take plural.

   Reply: This has been updated.

7. L527. ADAF: The acronym is not defined.

   Reply: We have now defined it on line 559 in the revised manuscript.

Reviewer 2's Comments:

The result is interesting and topical.

1. It is somewhat confusing to see ' 15 days' used throughout the main text, and also as the separation of the vertical lines in the figures, when the abstract - the first thing you read - states the quasi-period to be 17 days (albeit with large enough errors that 15 days is included). Even towards the end of the paper, the body of the paper is referring to 'the 15-day period'. This is really a stylistic / presentation issue.

   Reply: The peak in the periodogram occurs at 17(+1.2,-2.4) but visually, the individual X-ray peaks in Fig.1a align better with 15-d vertical lines. Given the uncertainty, it is possible that the value is close to 15-d and hence we refer to this signal as the 15-d quasi-periodicity throughout the manuscript. We have added text to lines 34-38 to clarify this aspect.

2. I think scientifically there the questions can fall in two groups : 1. Is the quasi-period real? 2. Is the interpretation correct?

   Is the signal real?

   My first thought on looking at the light curve in Fig 1a is that it doesn't really (at all) look like quasi-periodically spaced flares, but rather more (to me) like the long repeating sequences of X-ray behaviour seen in the X-ray binary GRS 1915+105 and a few other similar sources (I refer the authors to Belloni et al. A&A 355, 271 [2000] and Vincentelli et al. Nature 7950, 45, [2023]). In a sense these are quasi-periodic patterns of behaviour, which would show up in a Lomb-Scargle periodogram, but they are quite different from the type-C QPOs in XRBs which are widely inferred to be associated with Lense-Thirring precession (the authors' own reference [11], Ingram & Done]. Similarly, the patterns of oscillation in GRS 1915+105 and similar sources show temperature variations as seen here.

   More generally, having seen many thousands of light curves, I would not personally think you can confidently assert there is a quasi-period at all from those data, with so few cycles, half of which do not seem by eye to show a signal. That said, the authors state that it is statistically significant - I am not going to check their statistical methods, so we have to take this at face value.

   Reply: We agree with the referee that the existence of the quasi-periodicity in the lightcurve may not be immediately obvious by eye, and part of this likely arises from the fact that we do not have nearly as many cycles as are usually present in observations of X-ray binaries. However, we stress again that this is the nature of the predicted Lense-Thirring signal from tidal disruption events: a few cycles followed by termination (e.g., Stone and Loeb 2012: `https://arxiv.org/pdf/1109.6660.pdf`). This is because the TDE disc is expected to align with the black hole spin in a finite (and short) time. The duration of flares seen in AT2020ocn is comparable to

the predicted disk alignment timescale (please see Fig. 2 of Stone and Loeb 2012).

From a statistical analysis point, we have re-analyzed the data and re-produced the periodogram (which is now in the main body of the paper – see response to next point), and the signal is statistically significant for the range of continuum models we considered.

3. So maybe there is a signal, but I don't think it looks like the type-C QPOs in XRBs which are associated with L-T precession. As a side note, I also think the Lomb-Scargle periodogram should be part of the main paper, as it is central to the result, rather than in the additional material (which people often don't read).

   Reply: We thank the referee for the suggestion to move the periodogram to the main body of the paper – this has now been done.

   We agree with the referee that type-C QPOs in XRBs are typically interpreted as Lense-Thirring precession. However, due to the physical differences between XRBs and this accreting system (in particular the difference in black hole mass, the finite mass supply duration, and the radial extent of the precessing region), it is not clear to us that the precession should manifest in the same way. We discuss this in more detail in response to the comment below.

4. Is the interpretation correct?

   I think L-T precession is a possible interpretation of these data, but I do think the analogy with the quasi-periodically repeating state cycles in GRS 1915+105 is more natural at face value. That a SMBH should show disc instability patterns similar to stellar-mass BH and a NS is a pretty exciting result (and for me would be as interesting as the possible L-T interpretation and rather weak spin constraint [see below]).

   Reply: We thank the referee for drawing the analogy with some of the behavior exhibited by GRS 1915+105. We agree that it is possible that these two phenomena (the quasi-periodically repeating state cycles of GRS 1915+105, and the outbursts we have detected in AT2020ocn) are driven by the same physical process. In GRS 1915+105, this behavior has often been interpreted as evidence for the radiation pressure instability, but it has also been suggested that it may arise from Lense-Thirring precession of a radially narrow region of the disc close to the black hole horizon. We have now included a discussion of these points in the main text (lines 69-75).

   Also, please note that we have discussed the radiation pressure instability model and various other models in more detail in the Supplementary material, section 2.

5. I have to say up front that given there are so many uncertainties I do not think the spin can really be constrained. In XRBs the frequency of the type C QPO changes dramatically and in only one case, where the full set of three frequencies predicted by the relativistic precession model were present, was a BH spin estimate made. The authors themselves note that any spin estimate depends on a number of not-very-well-known parameters, but then they simply 'take standard parameters' or use estimated quantities (it's not very clear, for example, if the estimated error in the BH mass is propagated forward to the spin estimate).

Reply: We agree with the referee that the constraint on the black hole spin arises from a number of model-dependent assumptions. We have been up front about this in the paper, as the referee notes. To make it even more apparent, we have now added a sentence to the abstract paragraph. The range for the black hole spin that we quote from our model ($0.05 \lesssim |a| \lesssim 0.5$) does account for the uncertainty in the black hole mass ($10^{5.8}\, M_\odot$-$10^7\, M_\odot$) as well as a range of plausible disc density profiles ($-3/2 \leq s \leq 3/4$).

6. I am also curious - and I am sure this is just my ignorance - why we expect a L-T-generated QPO to dampen and disappear in TDEs as the disc aligns, whereas we don't see this in XRBs, where they persistent for millions of dynamical timescales?

   Reply: In XRBs, the material for accretion is provided by a companion, whose orbit may be misaligned with respect to the equatorial plane of the compact object. Hence the incoming material is also persistently misaligned and the accretion disc is subject to LT precession on a long-term basis. Thus it is likely that the material responsible for producing the QPO in XRBs would dampen and align on a relatively short timescale, but it is constantly replenished from a source of misaligned material and thus the effect can persist for many cycles. For TDEs around SMBHs, the fallback rate of material on to the disc decays strongly with time. Once the disc becomes geometrically thin (due to a decreasing accretion rate through the disc), the angular momentum of its inner parts quickly aligns with the spin of the SMBH due to the Bardeen-Petterson effect (warps then propagate on a viscous timescale that is longer than the local precession timescale, hence the disc does not behave as a rigid body anymore; Bardeen & Petterson 1975). This is expected to take a few months, and at this point LT precession ceases (please see e.g. Stone & Loeb 2012 `https://ui.adsabs.harvard.edu/abs/2012PhRvL.108f1302S/abstract` for details).

Reviewer Reports on the First Revision:

Referees' comments:

Referee #1 (Remarks to the Author):

The authors have now clarified satisfactorily all the points that I mentioned in my previous report.

Referee #2 (Remarks to the Author):

We thank the authors for taking into account our considerations and concerns. While I think some of the results remain uncertain, the discussion is much clearer about these uncertainties and is overall better presented.

It is still somewhat odd to mention a 17-day period in the abstract and have the rest of the paper refer to a 15-day period, I'd suggest you consider again changing that.

I think the paper will be of interest to a wide audience, and recommend publication.
